# Transcriptome architecture across tissues in the pig

**DOI:** 10.1186/1471-2164-9-173

**Published:** 2008-04-16

**Authors:** André LJ Ferraz, Ana Ojeda, Manel López-Béjar, Lana T Fernandes, Anna Castelló, Josep M Folch, Miguel Pérez-Enciso

**Affiliations:** 1 Departament de Ciència Animal i dels Aliments, Facultat de Veterinària, Universitat Autònoma de Barcelona, 08193 Bellaterra, Spain; 2 Faculdade de Ciências Agrárias e Veterinária, Universidade Estadual Paulista (UNESP), 14884-900 Jaboticabal – SP, Brazil; 3 Departament de Sanitat i d'Anatomia Animals, Facultat de Veterinària, Universitat Autònoma de Barcelona, 08193 Bellaterra, Spain; 4 Centre de Recerca en Sanitat Animal (CReSA), Universitat Autònoma de Barcelona, 08193 Bellaterra, Spain; 5 Institut Català de Recerca i Estudis Avançats (ICREA), C/Lluis Companys 23, 08010 Barcelona, Spain

## Abstract

**Background:**

Artificial selection has resulted in animal breeds with extreme phenotypes. As an organism is made up of many different tissues and organs, each with its own genetic programme, it is pertinent to ask: How relevant is tissue in terms of total transcriptome variability? Which are the genes most distinctly expressed between tissues? Does breed or sex equally affect the transcriptome across tissues?

**Results:**

In order to gain insight on these issues, we conducted microarray expression profiling of 16 different tissues from four animals of two extreme pig breeds, Large White and Iberian, two males and two females. Mixed model analysis and neighbor – joining trees showed that tissues with similar developmental origin clustered closer than those with different embryonic origins. Often a sound biological interpretation was possible for overrepresented gene ontology categories within differentially expressed genes between groups of tissues. For instance, an excess of nervous system or muscle development genes were found among tissues of ectoderm or mesoderm origins, respectively. Tissue accounted for ~11 times more variability than sex or breed. Nevertheless, we were able to confidently identify genes with differential expression across tissues between breeds (33 genes) and between sexes (19 genes). The genes primarily affected by sex were overall different than those affected by breed or tissue. Interaction with tissue can be important for differentially expressed genes between breeds but not so much for genes whose expression differ between sexes.

**Conclusion:**

Embryonic development leaves an enduring footprint on the transcriptome. The interaction in gene × tissue for differentially expressed genes between breeds suggests that animal breeding has targeted differentially each tissue's transcriptome.

## Background

It is now feasible to carry out large scale characterization of transcription activity using microarrays. This technology has opened new avenues to characterize and to dissect the transcriptome's genetic basis. It is a complementary approach to the classical ascertainment of the genetic architecture of complex traits, such as quantitative trait loci studies. There is now overwhelming evidence that the levels of mRNA are affected by a number of environmental, physiological and genetic factors, much the same as for any other quantitative, complex trait [1,2]. The extent and influence of each factor is, however, controversial and unknown to a large extent. Some authors reported that sex was far more important than the genetic line or age in *Drosophila *[3], whereas individual variability have been found to be very large in *Fundulus *fish [4]. The study of the genetic basis of variability at the transcriptome level is particularly relevant because, it has been claimed, evolution and thus phenotypic variability is due primarily to regulatory rather than structural mutations [4,5].

In many microarray studies so far, the goal has been to compare two physiological statuses, e.g., disease *vs*. healthy, by analyzing a single tissue in several individuals. Some studies have also compared different breeds or strains, again focussing on one or very few tissues in order to gain insight as to how much phenotypic variability correlates with differences at the transcriptome level; see for instance Reiner-Benaim et al. [6]. Nevertheless, the number of studies comparing different breeds is not very large yet, particularly in livestock. For instance, only four published studies have compared the global transcriptome of at least two porcine breeds [7-10] and one compared two divergent lines [11], whereas over a hundred dealing with porcine microarrays are indexed in *Pubmed*[12] as of November 2007. Thus, although a great deal is known about the transcriptome changes brought about by diseases, say cancer, much less is known about the relevance of genetic or environmental factors. Moreover, an organism is made up of many different tissues and organs, each with its own genetic programme. How different are these genetic programmes? How relevant are they in terms of total transcriptome variability? What are the relative contributions of breed or sex when assessed across tissues? Divergent livestock breeds offer extremely valuable genetic material to study these issues.

In order to contribute to answering these questions, here we study the variability in a large number of tissues pertaining to a reduced number of individuals. By allocating more experimental resources to a much larger number of tissues than usual, we aim at better characterizing the transcriptome variability across the whole organism. Although some studies have compared the transcriptome across different tissues [13-16], the contribution of each effect (e.g., tissue *vs*. sex) has not been quantified. The variability between tissues is not as well studied as that within tissues and some tissues remain poorly characterized, all the more in livestock species. The transcriptome of some tissues like liver and muscle have been analyzed extensively, well over a thousand citations in *Pubmed*[12] as of November 2007. Some other tissues have been only rarely studied despite their known physiological importance: only four microarray studies are reported for pineal gland, 19 for the hypophysis. Certainly, phenotypic differences in a given tissue can actually be due to changes that occur in a different organ. For instance, the fatness differences between individuals, a trait of utmost relevance in animal breeding, are more likely to be caused by genetic signals that originate in the hypothalamus or in the pineal gland rather than in the fat tissue itself.

The aim of this work was to study the global transcripome across tissues in two highly divergent pig breeds. We report a mixed model analysis of 16 tissues pertaining to four pigs, two Large White (LW) and two Iberian (IB), one male and one female per breed. These two breeds are phenotypically extreme for most traits of economic relevance, e.g., growth, fatness, reproductive performance. The tissues studied were olfactory bulb, hypothalamus, pineal gland, adenohypophysis, neurohypophysis, adrenal cortex, adrenal medulla, thyroid gland, diaphragm, *Biceps femoris *muscle, back fat tissue, abdominal fat tissue, stomach, liver, ileum and whole blood.

## Results and Discussion

### Transcriptome diversity across tissues and samples

**Figure 1:**
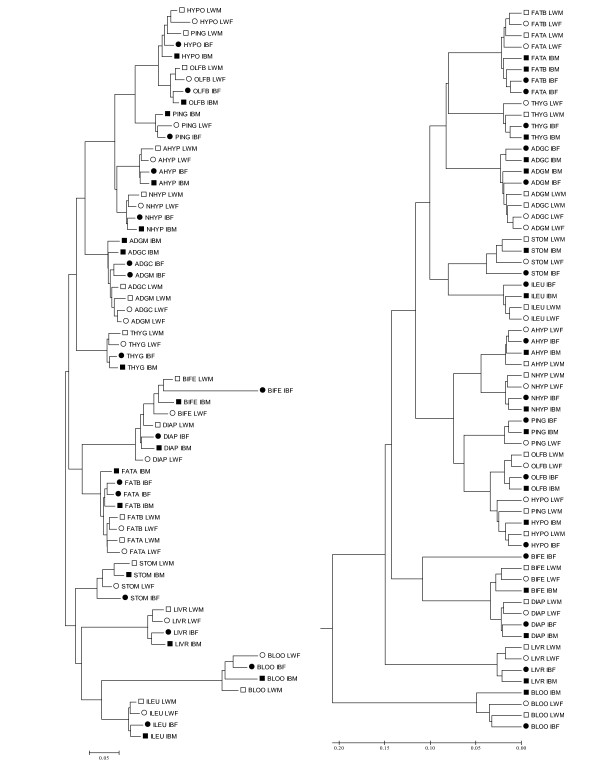
**NJ (left) and UPGMA trees (right) using the 1 - *r*^2 ^distance**. Each sample is named using the tissue acronym (four letters, Table 1), breed (LW or IB) and sex (M, male or F, female); LW males are indicated by open squares; LW females, by open circles; IB males, by black squares and IB females, by black circles.

Clustering is a useful starting exploratory tool to visualize highly dimensional data, and has been widely used to microarray data since the seminal paper of Eisen and cols. [17]. Here we applied two clustering methdos, the classical one based on the UPGMA criterion [17], and the neighbor-joing (NJ) clustering. In both cases, we used the distance one minus the squared correlation (1-r^2^) between the samples, after normalizing the raw data with the RMA procedure [18], as detailed in Material and Methods. Results are drawn in Figure 1, where it can be seen that samples were clearly grouped by tissue, next by breed in both trees. This was neatly observed for ileum, liver, thyroid gland, adeno and neurohypohysis and olfactory bulb. Muscle samples were clustered by tissue (diaphragma vs. M. *biceps femori*) but less clearly within each muscle. As for fat, the similarity was larger between tissues than between breeds, and samples of both back and abdominal fat origins were clustered together. The same was observed between cortex and medulla from adrenal gland. In this case, contamination between both tissues cannot be ruled out because of the irregular limits of the medulla that make not easy to separate that region neatly from the cortex collecting rapidly enough amount of tissue for analysis. Other authors have described previously contamination of medulla in the cortex sample when mechanical separation is performed[19]. Thus, this resemblance was not completely unexpected. The only outlier sample seemed to be the pineal gland of the Large White male (PING_LWM), which clustered with the rest of hypothalamus microarrays. Here contamination can be discarded in all likelihood because the two regions, hypothalamus and pineal gland, are in distinct areas of the brain. However, the pineal gland works in harmony with the hypothalamus. The former produces melatonin, which directly influences the function of various brain centers, including the hypothalamus. In stomach, less clearly in blood, samples were grouped by sex rather than by breed.

As expected, both UPGMA and NJ methods provided identical clustering at the first coalescence level. However, there were some interesting differences at higher levels. NJ identified four groups of tissues. The first group comprised brain tissues, including hypophysis; the second group, thyroid and adrenal endocrine glands; the third, muscle and fat tissues; whereas stomach, ileum, blood and liver were in the last group. In contrast, UPGMA clustered blood, liver and muscles in separate groups, while brain tissues were grouped together with fat, thyroid and adrenal glands; blood and liver were in distinct groups. It is well known that NJ have better properties than UPGMA when reconstructing evolutionary trees because it does not assume a constant evolutionary rate [20]. But is not evident how to translate this advantage in relation to the performance for classifying transcriptomes. The UPGMA method is equivalent to clustering algorithms implemented in popular microarray packages [17], whereas NJ have not been used so far, to our knowledge, in microarray studies. Here we found that, overall, NJ provided more biologically sound results than UPGMA: the first two groups in the NJ-tree comprised brain tissues and endocrine glands; muscle and fat tissues have a predominantly structural function in the body, while the rest of tissues (liver, stomach, ileum and blood) have an important metabolic role. In contrast, UPGMA results were not always so easily interpretable, e.g., it grouped hypophysis closer to fat tissues than to other brain organs like hypothalamus or the olfactory bulb.

**Figure 2:**
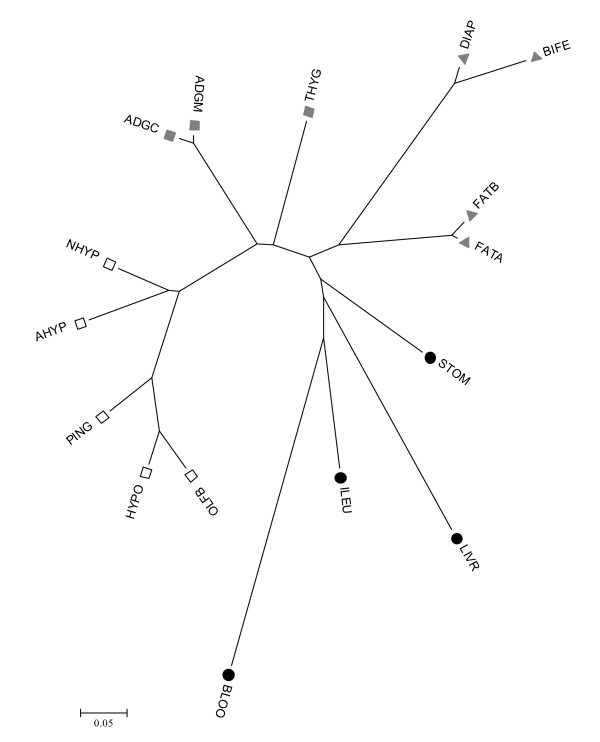
**NJ tree of tissues using the 1-$r_{T}^{2}$ Distance**. The four groups in Table 1 are indicated by symbols: brain (open squares), endocrine (grey squares), structural (grey triangles) and metabolic (black circles).

The trees depicted in Figure 1 were obtained with the original data (after RMA transformation). A better alternative to study relationships between tissues is to consider the *Probe × Tissue *solutions obtained from model (2), as these solutions are corrected for 'noisy' factors such as sex, breed and global tissue effects. Thus we constructed a NJ-tree of tissue transcriptomes using the 1-$r_{T}^{2}$ distance (see methods). This tree is shown in Figure 2. Although the pattern was similar to the NJ-tree in Figure 1, this tree provided a clearer picture of relationships between tissues. For instance, it emphasized that blood is the most distant tissue, corroborated also by the fact that blood harbored the largest number of extreme probes (Table 1). We defined a tissue's *extreme probe *as a probe for which all four mRNA levels of the tissue were either smaller or larger than the remaining mRNA levels (Material and Methods). In contrast, the two adrenal tissues and both fats were the closest pair of tissues. The brain is not a uniform organ, and this well known fact [21-23] was corroborated by a relevant variability within the different brain tissues sampled. Olfactory bulb was neighbor to hypothalamus. The two pituitaries, adenohypohysis and neurohypohysis, exhibited neighbor but distinct transcriptomes. This result agrees with the fact that both organs carry out very different physiological functions and have separate embryonic origins from the ectoderm layer (from an ectodermal outpocketing of the stomodeum and neural ectoderm, respectively). In fact, it was more interesting to note that neurohypophysis and hypothalamus were relatively distant. A close relationship might have been expected because neurohypophysis consists primarily of axonic projections of the hypothalamus. The pineal gland exhibited an intermediate transcriptome between hypophyses and hypothalamus – olfactory bulb.

**Table 1:** Tissues sampled

**Group**	**Tissue**	**Abbreviation**	**Embryonic origin**	**Function, remarks**	**Sampling details**	**# Extreme Probes^a^**	**Principal KEGG pathways of extreme probes**
Brain	Olfactory bulb	OLFB	Ectoderm (neural)	Perception of odors	Whole organ from both sides	274	Neuroactive ligand-receptor interaction WNT signalling pathway
	Hypothalamus	HYPT	Ectoderm (neural)	Regulates metabolic processes and other activities of the Autonomic Nervous System	Including mammilary body and grey tubercle	147	Neuroactive ligand-receptor interaction Cell adhesion molecules (CAMS)
	Pineal gland	PING	Ectoderm (neural)	Production of melatonin	Whole gland	89	-
	Adenohypophysis	AHYP	Ectoderm (stomodeum)	Hormone secretion regulating endocrine glands	Whole gland	241	Neuroactive ligand-receptor interaction
	Neurohypophysis	NHYP	Ectoderm (neural)	Store and liberation of hormones synthesized by the hypothalamus	Whole gland	130	WNT signalling pathway
Endocrine	Adrenal cortex	ADGC	Mesoderm	Synthesis of corticosteroid hormones	Part of the gland after mechanical separation	14	-
	Adrenal medulla	ADGM	Ectoderm (Neural crest)	Synthesis of adrenaline and noradrenalin	Part of the gland after mechanical separation	12	-
	Thyroid gland	THYG	Endoderm + Ectoderm (neural crest)	Production of thyroxine (T4), triiodothyronine (T3), and calcitonin	Part of the gland	376	-
Structural	Diaphragm muscle	DIAP	Mesoderm	Predominant oxidative metabolism	Samples from left and right crura	166	Oxidative phosphorylation ATP synthesis Glycolysis Citrate cycle Piruvate metabolism
	*M. Biceps femoris*	BIFE	Mesoderm	Glycolitic metabolism	Lateral area of the muscle	354	Glycolysis Insulin signalling pathway Frutose and manose metabolism Starch and sucrose metabolism Pentose phosphate pathway
	Back fat tissue	FATB	Mesoderm	Fat	Fat from lumbar region	51	-
	Abdominal fat tissue	FATA	Mesoderm	Fat	Fat external to the rectus sheath	21	-
Metabolic	Stomach	STOM	Endoderm	Digestion	Epithelium from the body	156	Tight junction
	Liver	LIVR	Endoderm	Central role in metabolism	Parts from quadrate and left lateral lobes	824	Urea cycle Bile acid biosynthesis Biosynthesis of steroids Complement and coagulation cascades Fatty acid metabolism Propanoate metabolism Amino acids metabolisms Butanoate metabolism Metabolism of xenobiotics by cytochrome P450
	Ileum	ILEU	Endoderm	Absorption of products of digestion	Epithelium	570	Cell cycle Ribosome Pyrimidine metabolism Antigen processing and presentation
	Blood	BLOO	Mesoderm	Supply of oxygen and nutrients	Blood from the femoral vein	3253	Hematopoietic cell lineage Regulation of actin cytoskeleton T-cell receptor signalling Focal adhesion ECM receptor interaction Cytokine cytokine receptor interaction Cell adhesion molecules (CAMS) MAPK signalling pathway JAK-STAT signalling pathway Tight junction

^a ^The complete list is in Supplementary Table S2. See Material and Methods for definition of extreme probe.

Interestingly, there was a relation between embryonic origin and clustering. Most tissues with the same embryonic origin clustered together in the NJ-tree (Figure 2). All brain tissues are of ectodermic origin, as is hypophysis, and these tissues were clustered together in the first group of tissues. The second group comprised the adrenal and thyroid glands, tissues with double embryonic origin. The adrenal medulla derives from ectoderm (neural crest cells), while the cortex develops from mesoderm. The thyroid gland develops from two distinct embryonic lineages: follicular cells (which produce thyroxine) and parafollicular C-cells (which produce calcitonin) and are of endodermal and ectodermal neural crest origins, respectively [24]. Mesoderm gives origin to fat and muscle. Liver, and stomach and ileum epithelia are derived from endoderm. Finally, blood, the most separate and outlier tissue, is of mesoderm origin. Thus, it seems that embryonic development leaves a standing footprint on the transcriptome in each of the tissues.

### Functional analysis of extreme genes across embryo origins and tissues

**Figure 3:**
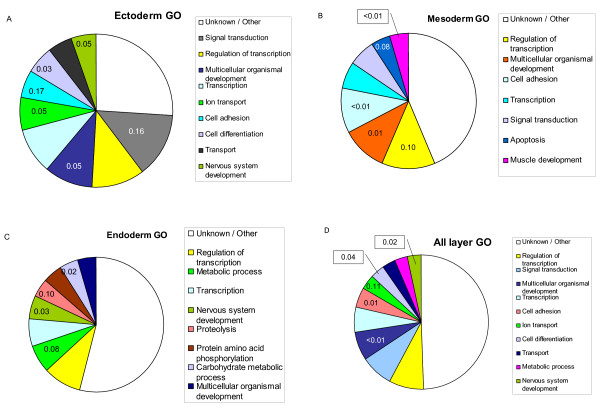
**Differential GO categories across embryo layers**. Percentage of the most frequent GO categories within extreme genes for each embryonic layer (**A**, ectoderm; **B**, mesoderm; **C**, endoderm; **D**, all genes in **A, B **and **C**). The number in each category is the false discovery rate (FDR) that the category is over represented with respect to the GO frequency across all genes in the microarray. The FDR is shown only if < 0.20.

We sought to investigate more in detail the genetic basis of the tissue arrangement by embryonic layers, and to pick up genes that can be differentially expressed in concerted action within each embryonic layer. To that end, we obtained the extreme gene probes differentiating the ectoderm tissues (olfactory bulb, hypothalamus, pineal gland, adenohypophysis and neurohypophysis), mesoderm (muscle and fat), and endoderm (liver, stomach and ileum). An extreme probe for each embryo layer was defined as for individual tissues, i.e., a probe whose all expression levels for that group of tissues were either smaller or larger than the levels for the rest of tissues (Material and Methods). We excluded thyroid and adrenal glands for being mixed tissues and blood, for outlier. The complete list in additional file 1. A pie-plot with differential gene ontology (GO) frequencies obtained with onto-express [25] is in Figure 3. A wide variety of GO were over represented in each layer; this fact, together with a large percentage of unknown or unannotated genes makes it somewhat difficult to interpret the results in detail.

Neverheless, some interesting results appeared. For instance, genes with ontologies like nervous development and ion transport – clearly related to central nervous system – were more frequent than expected within ectoderm extreme genes, as were genes involved in development (Figure 3A). In particular, some of the most overexpressed genes like INA (internexin neuronal intermediate filament protein), ASTN (astrotactin) or NAPIL2 (nucleosome assembly protein 1-like 2) are involved in neuron development, whereas others like GABRG2 (GABA A receptor) or SYT4 (synaptotagmin IV) are involved in normal neuron functioning. The case of NAP1L2 (nucleosome assembly protein 1-like 2) is particularly interesting. Although, according to *Pubmed*, 'the function of this family member is unknown, mouse studies suggest that it represents a class of tissue-specific factors interacting with chromatin to regulate neuronal cell proliferation'. Our results strongly suggest that NAP1L2 is involved in neuronal system and that the function is maintained across species.

In parallel to results for ecctoderm tissues, the most significant enriched ontology was muscle development in the mesoderm (Figure 3B). Here, some of the most extreme genes were Hox genes involved in limb development and myogenesis (e.g., PRRX1, HOXD8 and MEOX2). In particular, it has been suggested that PRRX1 and HOXD8 are also involved in the urogenital tract development, also of mesoderm origin. The two top extreme genes for mesoderm tissues gamma-sarcoglycans (SGCD and SGCG) are also involved in muscle development. As for the endoderm (liver, ileum and stomach), an excess of genes involved in general metabolism was observed, although we also found a significant enrichment in nervous development genes (Figure 3C). Two of the most overexpressed extreme genes, HNF4A (hepatocyte nuclear factor 4, alpha) and FOXA3 (hepatocyte nuclear factor 3 or forkhead box A3) are known to be involved in liver development, and it is suspected that HNF4A can play a role also in intestine development, which is supported by our data. Intriguingly, some of the most extreme overexpressed genes were involved in the complement pathway (C5, BF, SDC1, C4BPA), may be as a consequence of the defense function of the ileum. Finally, when all extreme genes across the three embryo layers were considered jointly (Figure 3D), the most significant ontologies were nervous system development, cell differentiation, cell adhesion and multicellular organism development, which again suggests a sound biological interpretation.

We also obtained the extreme probes for each of the individual tissues. The main over represented biological process per tissue is in Table 1, and a complete list of the genes is in additional file 2. Results confirmed, overall, previous biological knowledge. brain tissues (olfactory bulb, hypophyses, pineal gland and hypothalamus) exhibited a large number of genes in signalling pathways (see also Figure 3A). The highly oxidative muscle diaphragma was enriched in genes involved in ATP synthesis through citrate cycle and oxidative phosphorylation, whereas *biceps femori *(glycolytic muscle) extreme genes were often related to the production of ATP by glycolysis and sugar pathway. The liver extreme genes were in the urea cycle pathway and bile acid and amino acid metabolisms; blood genes pertained to hematopoietic processes and signaling pathway systems.

### Mixed model analysis

Mixed model analyses were highly successful in modelling transcriptome variability (Table 2). For instance, for model (2) the fraction of unexplained variance was very small, 1 - $h_{P}^{2}$ - $h_{PT}^{2}$ = 0.03, and ~15% in the rest of models. This means that the set of expression levels can be very accurately modelled as a random normal variate in the log scale. Note that models (1 – 4) were highly parsimonious: a few parameters, i.e., the variance components and the fixed effects tissue, sex and breed were needed to model the data. By far, the largest variance component was that of the probe, which explained overall at least 85% of the total variability (Table 2). The interaction probe × tissue ($\sigma_{PT}^{2}$) accounted for 11% of phenotypic variance (model 2), whereas the total variances explained by probe × breed or probe × sex were marginal, only about 1% (models 3 and 4). These were the *global *variance estimates, when all probes were considered jointly. It does not follow that sex or breed were not associated to changes in gene expression. The relative importance of sex or breed did increase when we restricted the analysis to a data subset. For instance, the percentage of total variance explained by breed or sex increased to 25% or 48% when we used the 100 most differentially expressed genes between breeds or between sexes, respectively (last two rows of Table 2). Symmetrically, the relevance of probe effect decreased for these data subsets.

**Table 2:** Parameter estimates

**Model**	**Dataset**	$h_{P}^{2}$	$h_{PT}^{2}$	$h_{PB}^{2}$	$h_{PS}^{2}$
**1**	Complete	0.87	-	-	-
**2**	Complete	0.86	0.11	-	-
**3**	Complete	0.85	-	0.01	-
**4**	Complete	0.85	-	-	0.01
**3**	100 largest z^b^	0.46	-	0.25	-
**4**	100 largest z^s^	0.25	-	-	0.48

### Differential gene expression

**Figure 4:**
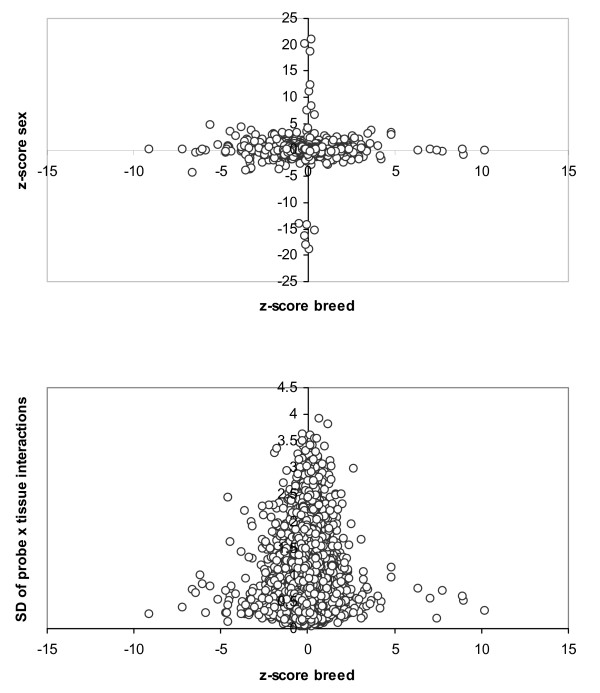
**Relation between *z*-scores**. Plot of *z*-scores of breeds and sexes (top), and between the breed *z*-scores and the standard deviation within probes of *Probe × Tissue *solutions (bottom).

At a false discovery rate FDR < 0.05, we identified 19 and 33 genes differentially expressed between sexes and breeds, respectively. The complete lists together with tentative annotations [26] are in Tables 3 and 4. Two of the genes, orthologous to human genes in the HLA-A and HLA-B complex, were differentially expressed simultaneously between breeds and between sexes. However, there was no correlation overall between tissue, breed or sex effects. This was evident from the pattern in Figure 4, which displays the relation between *z*^*b *^and *z*^*s *^scores (top figure) and between *z*^*b *^scores and the standard deviation of the probe × tissue solutions (bottom figure). If there were a relation, we would see the dots around a diagonal rather than close to the axes. Thus, each of the factors studied here, sex and breed, influence the transcriptome through different genetic programmes.

**Table 3:** Differentially expressed genes between sexes at FDR < 0.05

Gene	Symbol	Probe	*z*-score	Function, GO-process
DEAD (Asp-Glu-Ala-Asp) box polypeptide 3, Y-linked	DDX3Y	Ssc.7473.1.A1_at	20.96	Nucleoside-triphosphatase activity
Eukaryotic translation initiation factor 2, subunit 3 gamma,	EIF2S3	Ssc.16426.1.S1_at	20.19	Protein metabolism/translation regulator activity
FAM5C – family with sequence similarity 5, member C	FAM5C	Ssc.6667.1.A1_at	-18.80	Unknown
Eukaryotic translation initiation factor 1A, Y-linked	EIF1AY	Ssc.26799.1.S1_at	18.77	Translational initiation/translation regulator activity
DENN/MADD domain containing 4A	DENND4A	Ssc.13426.1.A1_at	-18.07	Regulation of transcription, DNA-dependent
Protein tyrosine phosphatase, receptor type, M	PTPRM	Ssc.31029.1.A1_at	-16.39	Protein amino acid dephosphorylation/protein tyrosine phosphatase activity
Latrophilin 2	LPHN2	Ssc.21512.1.A1_at	-15.40	Neuropeptide signaling pathway/signal transducer activity
Homeodomain interacting protein kinase 2	HIPK2	Ssc.2434.1.A1_at	-14.25	Positive regulation of programmed cell death/transcription regulator activity
Circadian locomoter output cycles kaput; clock homolog	CLOCK	Ssc.4897.1.A1_at	-14.10	Circadian rhythm/transcription regulator activity
Thymosin, beta 4, X-linked	TMSB4X	Ssc.27304.1.S1_at	12.26	Cytoskeleton organization and biogenesis/actin binding
Ubiquitously transcribed tetratricopeptide repeat gene, Y-linked	UTY	Ssc.27236.1.S1_at	11.10	Unknown. Positive regulation of growth rate (*C. elegans*)
Jumonji, AT rich interactive domain 1C	JARID1C	Ssc.21814.1.S1_at	8.41	Regulation of transcription, DNA-dependent/oxidoreductase activity
Ubiquitously transcribed tetratricopeptide repeat, X chromosome	UTX	Ssc.15821.1.S1_at	7.45	Unknown. Positive regulation of growth rate (*C. elegans*)
Glutathione S-transferase A1	GSTA1	Ssc.16377.1.A1_at	6.67	Glutathione metabolism/transferase activity
Major histocompatibility complex, class I, A	HLA-A	Ssc.13780.11.S1_x_at	4.81	Antigen processing, endogenous antigen via MHC class I/MHC class I receptor activity
Major histocompatibility complex, class I, B	HLA-B	Ssc.18552.1.S1_at	4.39	Antigen processing, endogenous antigen via MHC class I/MHC class I receptor activity
Major histocompatibility complex, class I, B	HLA-B	Ssc.18554.1.S1_x_at	-4.38	Antigen processing, endogenous antigen via MHC class I/
Zinc finger protein, X-linked	ZFX	Ssc.26228.1.S1_at	4.19	Regulation of transcription/transcriptional activator activity
Hypothetical protein FLJ20035	Q8IY21	Ssc.18924.1.A1_at	-3.95	Unknown

*z *– scores correspond to male minus female contrasts.

**Table 4:** Differentially expressed genes between breeds at FDR < 0.05

Gene	Symbol	Probe	z-score	Function, GO-process
Prostaglandin E synthase 2	PTGS2	Ssc.25850.1.A1_at	10.20	Regulation of inflammatory response/prostaglandin-endoperoxide synthase activity
Hypothetical protein DKFZp313A2432	Q8NHG7	Ssc.29609.2.A1_at	- 9.11	Unknown
ATPase, H+ transporting, lysosomal 31 kDa, V1 subunit E1	ATP6V1G2	Ssc.12005.1.A1_at	8.97	Purine ribonucleotide metabolism/ion transporter activity
Synovial sarcoma, X breakpoint 2 interacting protein	SSX2IP	Ssc.28283.1.A1_at	8.89	Cell adhesion
Sema domain, immunoglobulin domain (Ig), short basic domain, secreted, (semaphorin) 3A	SEMA3A	Ssc.29388.1.A1_at	7.74	Cell differentiation (nervous system development)
DIDO1 death inducer-obliterator 1	DATF1	Ssc.14412.1.A1_at	7.46	Apoptosis (*M. musculus*)/regulation of transcription, DNA-dependent
Hypothetical protein DKFZp313A2432	Q8NHG7	Ssc.29609.1.S1_at	- 7.18	Unknown
Immunoglobulin heavy constant mu	IGHM	Ssc.7706.1.A1_at	7.09	Response to biotic stimulus/signal transducer activity
Major histocompatibility complex, class I, B	HLA-B	Ssc.18554.1.S1_x_at	- 6.61	Antigen processing, endogenous antigen via MHC class I/MHC class I receptor activity
Armadillo repeat containing, X-linked 1	ARMCX1	Ssc.5616.1.S1_at	- 6.42	Cellular component
Tousled-like kinase 2	TLK2	Ssc.30422.1.A1_at	6.36	Establishment and/or maintenance of chromatin architecture/transferase activity, transferring phosphorus-containing groups
Follistatin-like 4	FSTL4	Ssc.11743.1.S1_at	- 6.15	Calcium ion binding
Hypothetical protein	Q8N5E3	Ssc.1256.1.A1_at	- 6.03	Unknwon
Transcription factor CP2	TFCP2	Ssc.7954.1.A1_at	- 5.83	Regulation of nucleic acid metabolism/transcription regulator activity
Major histocompatibility complex, class I, A	HLA-A	Ssc.13780.11.S1_x_at	- 5.60	Antigen processing, endogenous antigen via MHC class I/MHC class I receptor activity
Golgi phosphoprotein 4	GOLPH4	Ssc.25176.1.A1_at	- 5.12	Cellular component
Enoyl Coenzyme A hydratase domain containing 1	ECHDC1	Ssc.1146.1.S1_at	4.86	Metabolism/catalytic activity
Glycoprotein M6B	GPM6B	Ssc.8133.1.A1_at	4.85	Cell differentiation (nervous system development)/molecular function unknown
Hypothetical protein	C21orf88	Ssc.22421.1.A1_at	- 4.68	Unknwon
Polo-like kinase 2	PLK2	Ssc.29934.1.A1_at	- 4.67	Positive regulation of signal transduction/transferase activity, transferring phosphorus-containing groups
Hypothetical protein	Q9NV98	Ssc.5839.2.A1_at	- 4.67	Unknwon
Lysozyme	LYZ	Ssc.670.1.S1_at	- 4.58	Cellular catabolism (cell death)/hydrolase activity, acting on glycosyl bonds
RAB18, member RAS oncogene family	RAB18	Ssc.30567.1.A1_at	- 4.56	Endocytosis/pyrophosphatase activity
Zinc finger protein 12	ZNF12	Ssc.10665.1.A1_at	- 4.56	Regulation of cellular process/zinc ion binding
Splicing factor, arginine/serine-rich 2, interacting protein	SFRS2IP	Ssc.12708.1.A1_at	- 4.50	mRNA processing
Immunoglobulin heavy constant mu	IGHM	Ssc.11070.1.S1_at	- 4.46	Response to biotic stimulus/signal transducer activity
Synapsin III	SYN3	Ssc.3880.1.S1_at	-4.43	Regulation of neurotransmitter levels (transmission of nerve impulse)/purine nucleotide binding
Deleted in liver cancer 1	DLC1	Ssc.18150.1.A1_at	- 4.40	Negative regulation of growth/enzyme regulator activity
DEAD (Asp-Glu-Ala-Asp) box polypeptide 17	DDX17	Ssc.8674.1.A1_at	4.24	Nucleobase, nucleoside, nucleotide and nucleic acid metabolism/nucleoside-triphosphatase activity
Potassium channel modulatory factor 1	KCMF1	Ssc.25963.1.A1_at	4.16	Channel or pore class transporter activity
Mitochondrial carrier homolog 2	MTCH2	Ssc.6054.2.S1_at	- 4.12	Transport
Cytoskeleton-associated protein 4	CKAP4	Ssc.2147.2.A1_at	4.09	Cellular component
Blood vessel epicardial substance	BVES	Ssc.15540.1.A1_at	- 3.82	Muscle development

z – scores correspond to Large White minus Iberian contrasts.

**Figure 5:**
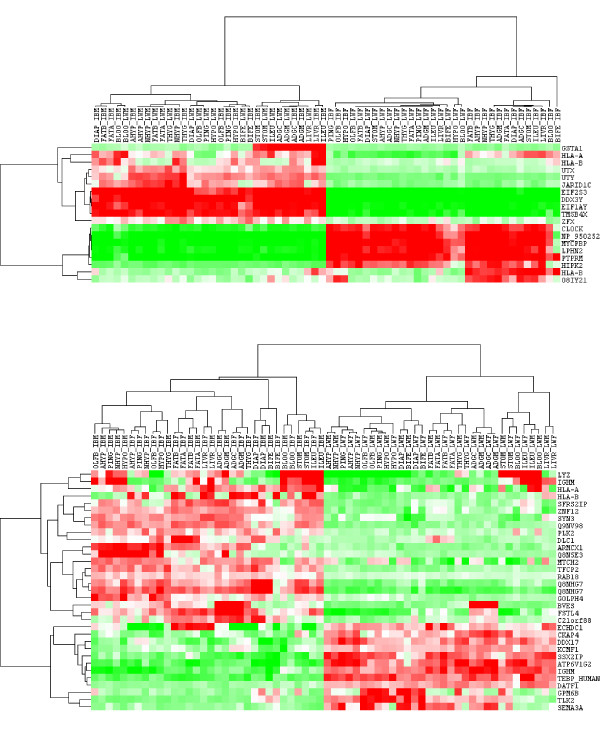
**Sample clustering using differentially expressed genes**. Genes differentially expressed between sexes (top) and breeds (bottom).

We performed a clustering of probes and samples using only the differentially expressed genes from Tables 3 and 4. Results were drawn in Figure 5, where the discrimination between sexes (top) and breeds (bottom) is neat and clearly visible. This clustering contrasts with that in Figure 1 where all probes were employed. The discrepancy occurs because the expression pattern of most genes is primarily affected by the tissue and not so much by sex or breed, as discussed. However, as Figure 5 clearly shows, this does not mean that a careful selection of probes does not allow us to discriminate samples between according to sex or breed.

The list of differentially expressed genes between sexes comprised eight X or Y linked genes, based on the localization of orthologous human genes (Table 3). Note that three X-linked genes were up-regulated in males (ZFX, UTX and TMSB4X), a phenomenon also observed by Yang et al. [14] although for different genes. Male up-regulated genes were predominantly involved in spermatogenesis (DDX3Y), cell proliferation, migration, and differentiation (GSTA1, TMSB4X), as well as genes genes from the histocompatiblity complex (HLA-A, HLA-B). Female up-regulated genes had functions related to positive regulation of the transforming growth factor beta receptor signaling pathway (HIPK2), proteins up-regulated in gonadotropinomas (FAM5C) or affecting the circadian rhytm (CLOCK). Up-regulation of CLOCK in females is consistent with evidence showing that this gene is related to the cyclic nature of the reproductive process in females, both in mammal and in non mammal species [27-29].

We found a good concordance with the list of differentially expressed genes obtained with the same microarray in lung and mesenteric lymph node by our group in a separate experiment (Fernandes *et al*., unpublished results). Thirteen genes (mesenteric lymph node) and ten genes (lung) were also in Table 3, despite the fact that corresponded to different tissues, treatments and breeds. Yang et al. [14] compared microarrays of 169 female and 165 male mice in four tissues (whole brain, liver, skeletal muscle and gonadal adipose), the largest study to date of sex differentially expressed genes. They reported 27 genes that were differentially expressed in all four tissues, four of them (DDX3Y, EIF2S3Y, JARID1D and UTX) were also found here; DDX3Y and EIF2S3Y were the most differentially expressed genes found here and in [14]. It is interesting to note that these authors also reported that immune genes, e.g., histocompatiblity genes, were overexpressed in males with respect to females, in agreement again with our results [14,30]. Differential expression between sexes for SSCY genes can be considered as validated.

We also validated probe (Ssc.4897.1.A1_at), annotated as CLOCK gene [26], by real time quantitative RT-PCR (QRT-PCR) in backfat samples of 27 pigs. QRT-PCR results clearly confirmed the microarray data (Table 5). The average relative expression was 1.88 ± 0.68 in females, whereas we were not able to reliably detect expression in males at 1/2000 cDNA dilution because we were below the limit of detection. To verify whether this gene product is actually expressed in males, we repeated the assay at 1/20 cDNA dilution. We could detect low levels of expression in four out of the 13 males studied. Iberian male 27, that with largest relative quantification (RQ), exhibited nonetheless a expression level ~100 fold lower than in females.

**Table 5:** QRT-PCR results for CLOCK gene, probe Ssc.4897.1.A1_at

**Sample**	**Sex**	**Breed**	**RQ (1/2000)**	**RQ (1/20)**
13	F	Duroc	1.000	NA
14	F	Duroc	1.555	NA
16	F	Duroc	2.694	NA
22	F	Iberian	1.114	NA
25	F	Iberian	2.054	NA
26	F	Iberian	1.581	NA
8	F	Landrace	1.621	NA
10	F	Landrace	1.521	NA
1	F	Large White	1.272	NA
3	F	Large White	1.796	NA
5	F	Large White	2.696	NA
6	F	Large White	1.961	NA
17	F	Synthetic	1.951	NA
21	F	Synthetic	3.482	NA
12	M	Duroc	BLD	BLD
15	M	Duroc	BLD	BLD
23	M	Iberian	BLD	BLD
24	M	Iberian	BLD	0.012
27	M	Iberian	BLD	0.010
7	M	Landrace	BLD	BLD
9	M	Landrace	BLD	BLD
11	M	Landrace	BLD	0.001
2	M	Large White	BLD	0.003
4	M	Large White	BLD	BLD
18	M	Synthetic	BLD	BLD
19	M	Synthetic	BLD	BLD
20	M	Synthetic	BLD	BLD

Sex: F, female; M, male

RQ (1/2000): Relative quantification RQ, average of three replicates each, at 1/2000 cDNA dilution. The results are given in terms of sample number 13, used as calibrator, i.e., RQ = 1

RQ (1/20): Relative quantification RQ, average of three replicates each, at 1/20 cDNA dilution, only analyzed for males

BLD, below limit of detection

NA, not available

The list of probe sets corresponding to differentially expressed genes at FDR < 0.05 between LW and IB breeds is in Table 4. The thirty three probes corresponded to 27 genes and 5 hypothetical proteins; 12 were over expressed in Large White (LW-biased) and 21 over expressed in Iberian (IB-biased). The LW-biased list comprised genes involved in ATP biosynthetic process, nervous system development, lipid metabolism, RNA processing, and control of growing and cellular division. The IB-biased genes affected the regulation of cell growth, RNA splicing factor activity or muscle development. These results hint that transcriptome differences between breeds seem to be manifold. No particular single biological process was predominantly affected. It is to be noticed, nonetheless, that there were several genes involved in nervous development (SEMA3A, GPM6B, SYN3) or growth and cell cycle (DATF1, LYZ, RAB18, ZNF12, DLC1, BVES). As there is currently very little information on the transcriptome differences between pig breeds, it is not possible to know how general these results will be. Some recent results have compared muscle in Duroc vs. Taoyuan breeds [8], in Landrace vs. Tongcheng [10] and liver and muscle in Duroc vs. Pietrain [7,9]. While these studies have focussed on a particular tissue and with different techniques and microarrays, all suggest that breeds differ in genes related to a variety of functional categories. In some cases, the microarray included predominantly genes involved in muscle structure and development or energy metabolism [7] so their results may not be representative of the whole genome.

It should be noted that the differentially expressed genes reported here (Tables 3 and 4) were those that exhibited largest differential expression *across *all tissues. This follows from the specification of models (1–4), where all probes were analyzed simultaneously and where the tissue effect was included in the models as just an additional effect. Thus, it is important to bear in mind that other genes could show larger differential expressions in a specific tissue than those listed in Tables 3 and 4. The experimental design of this study did not allow us to characterize with reasonable FDR the specific expression of a gene in a single tissue. Nevertheless, we can get a glimpse of the effect of tissue in differential expression by subdividing the tissues in groups. We reasoned that the probe × tissue interaction should be larger between tissues that clustered far apart, as their genetic programmes are more different that tissues that are very similar, e.g., back and abdominal fats. We defined the following tissue groups base on the NJ-tree shown in Figure 2: brain (olfactory bulb, hypophises, hypothalamus and pineal gland), endocrine (adrenal and thyroid glands), structural (fat and muscle) and metabolic tissues (stomach, ileum, liver and blood). To a large extent, these groups reflect also a common embryonic origin (Table 1).

We reanalyzed each of the groups with models 3 and 4, obtaining new *z*^*b *^and *z*^*s *^scores for each of the tissue groups. Table 6 presents the correlation between the z-scores obtained with all tissues analyzed simultaneously and each of the groups. In order to focus on the most relevant genes, the correlations shown were those obtained with the 100 genes with largest absolute *z*-scores. The pattern shown was highly illuminating. For sex, there seemed to be little interaction, as *z*^*s*^-scores were highly correlated across groups of tissues. Correlation coefficients were always > 0.9. In contrast, correlations between *z*^*b *^scores were much more variable and, importantly, much lower overall. Thus, whereas the correlation between brain and metabolic tissues was 0.91 for sex *z*-scores, it was only 0.34 for breed *z*-scores. This means that most differentially expressed genes between sexes were shared across tissues whereas this was much more unlikely when comparing two distant breeds like Iberian and Large White. It is interesting to note that the clusters in Figure 5 also hinted that heterogeneity was larger within breed differentially expressed genes than within sex differentially expressed genes. Note that the color patterns of sex differentially expressed genes were more uniform across samples than for breed differentially expressed genes. Besides, clustering was much 'flatter' – *i.e*., more uniform – within sexes than within breeds. This result may have important implications. It suggests that physiological changes responsible for breed differences have targeted differentially the transcriptome across tissues. Not all tissues have been equally affected. It remains to be studied on which of the tissues the effect of breed differentiation has been the largest. Our current data set does not allow us to respond to this question accurately: the probe × tissue heritabilities ($h_{PB}^{2}$) were very similar in all tissue groups and no clear pattern emerged from Figure 1.

**Table 6:** Correlation between between *z*-scores in different tissue groups

	All	Brain	Endocrine	Structural	Metabolic
All	-	0.98	0.97	0.98	0.97
Brain	0.79	-	0.92	0.96	0.91
Endocrine	0.87	0.62	-	0.92	0.97
Structural	0.83	0.46	0.68	-	0.93
Metabolic	0.77	0.34	0.63	0.64	-

The tissues included in each group are listed in Table 1. The *z*-scores correspond to the 100 largest differentially expressed genes when all tissues are jointly analyzed. Upper diagonal, sex differentially expressed genes. Lower diagonal, breed differentially expressed genes.

### Functional annotation of sex and breed differentially expressed genes

**Figure 6:**
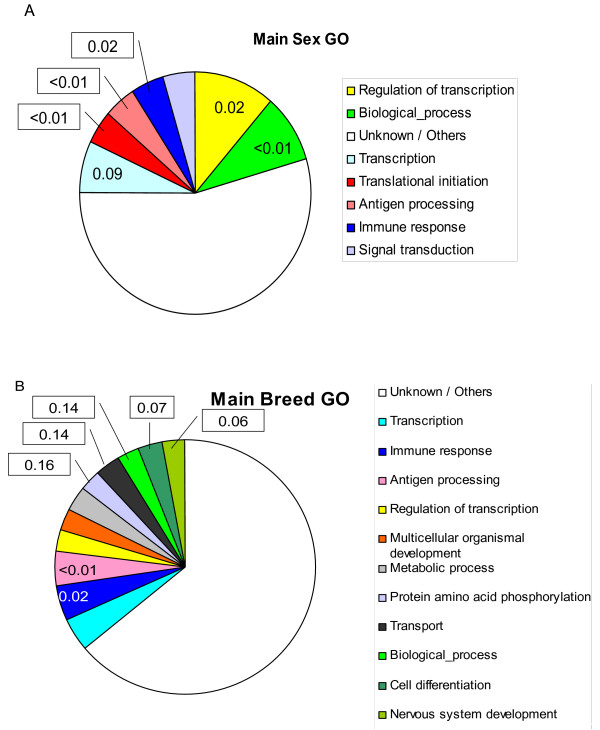
**Proportion of functional annotation categories**. Percentage of the most frequent GO categories within the most significant differentially expressed genes between sexes (Table 3) and between breeds (Table 4). The number in each category is the false discovery rate (FDR) that the category is over represented with respect to the GO frequency across all genes in the microarray. The FDR is shown only if < 0.20.

We carried a GO automatic annotation [25] with the 19 most differentially expressed genes between sexes and 33 between breeds. The corresponding plots are in Figure 6. In neither case, sex or breed, was a given biological process GO clearly over represented (except the class of *unknown/others *which pools unknown and minoritary GO classes). As a result, the discussion should be considered as tentative or provisional. Nevertheless, we found defense genes to be over represented for both sex and breed specific genes. For sex, there was a significant excess of transcription and translation related genes. Some of these genes are sex linked (EIF1AY, UTX, ZFX), as mentioned above, so an over representation of this ontology is not unexpected. The GO biological processes were more scattered for breed than for sex (Figure 6B vs. 6A). Interestingly, there were more genes involved in nervous system development and cell differentiation than expected among breed specific genes. This might provide some clues as to what are the primary changes exerted by selection and breeding in the organism' transcriptome but further work is needed to get a definitive answer.

## Global Discussion and Conclusion

This study shows that a much larger fraction of transcriptome variability is due to tissue differences rather than to sex or breed. Our results agree with recent literature [13,15] that showed that transcriptional co-expression is indicative of gene function and that tissues with similar function tend to cluster together. An additional relevant observation from the NJ-clustering (Figures 1 and 2) was that embryonic development seems to leave an enduring footprint on the transcriptome: tissues with similar developmental origin tend to cluster closer than those with different embryonic origins. As clustering was carried out with all probes simultaneously, this means that the footprint extends to the majority of genes. We further characterized the GO biological process of the extreme genes between tissues or groups of tissues sharing a common embryonic origin (Figure 3, and additional files 1 and 2). We found that, often, a sound biological interpretation was possible. For instance, when all extreme genes across the three embryo layers were considered jointly (Figure 3D), the most frequent and significant ontologies were related to development. We also indentified several genes involved in tissue development that have a distinct expression pattern in tissues with a common embryonic origin. This distinct expression pattern is maintained beyond embryo development.

A key element of our analysis was mixed model methods. Although well known to statisticians and animal breeders [31], these techniques are not so widely employed to dissect microarray data. Nevertheless, its use have been advocated and successfully employed in a number of studies, e.g., [32,33]. We report that mixed modeling explained a large part of total variability, with a very parsimonious parameterization. A relevant advantage over standard methods is that all data are analyzed simultaneously as opposed to more utilized methods like ANOVA – based techniques or bioconductor's package *limma *[34]; as a consequence, reduced standard errors are expected. In a second step, one can use the solutions from the models to carry out clustering or differential expression studies, as we did here.

Although the variance explained by the effect of tissue was much larger (~11 times) than that explained by sex or breed, which were comparable (Table 2), we were able to identify genes with differential expression between breeds and between sexes. Noticeably, different genes were affected by each factor, i.e., the genes primarily affected by sex were different than those affected by breed or tissue. Are genes whose expression differ between breeds differentially expressed across all tissues or is there any interaction tissue × gene? Our experimental design does not allow to resolve this issue with enough power. However, the results (Figure 5, Table 6) hint that interaction can be important for differentially expressed genes between breeds but not so much between sexes. Resolving this question is of the utmost interest, both to understand the profound influences of artificial selection on the organisms and to propose novel more efficient strategies for animal breeding.

Although more work is needed to calibrate the actual relevance of breed or genetic differences to the pig's transcriptome (or in any other species), it seems difficult that the importance of breed or sex differences exceeds that of tissue. Thus, the argument that regulatory rather than structural mutations are a more important source of phenotypic variability [5] needs then to be considered in perspective. It follows from our study that differences within the same individual largely exceeds those between individuals.

## Methods

### Animal material

Four animals, two Large White (LW) and two Iberian (IB) piglets were bought from two breeding companies and transferred to the University experimental farms at weaning, i.e., aged one month approximately. Pigs were housed simultaneously, fed the same diets during the fattening period, that lasted two months, and were weighed at weekly intervals. At the time of slaughter, the average ages were 87 and 89 days for LW and IB, respectively. Their mean live weights at that time were 37.5 (LW) and 29.1 kg (IB). The four animals were identified as LW or IB and male (M) or female (F), *i.e*., LWM, LWF, IBM and IBF.

Animals were euthanized by an overdose of intravenous sodium thiobarbital. At necropsy, samples of 16 tissues were collected, snap frozen in liquid nitrogen and stored at -80°C. The average time gap between euthanasia and tissue collection was ~15 minutes, maximum time was 25 minutes. The tissues collected were olfactory bulb (OLFB), hypothalamus (HYPO), pineal gland (PING), adenohypophysis (AHYP), neurohypophysis (NHYP), adrenal cortex (ADGC), adrenal medulla (ADGM), thyroid gland (THYG), diaphragm (DIAP), M. *Biceps femoris *(BIFE), back fat tissue (FATB), abdominal fat tissue (FATA), stomach (STOM), liver (LIVR), ileum (ILEU) and whole blood (BLOO). Adrenal cortex and medulla were separated by a sharp knife. The hypothalamus included the mamillary body and grey tubercle but excluded the chiasma opticum. The nomenclature for organs and tissues was used according to the *Nomina Anatomica Veterinaria*[35]. More details are given in Table 1. Throughout this work, a sample is identified by the acronym of the tissue followed by the animal id, e.g., FATB_LWF refers to back fat tissue from the female Large White. All procedures were approved by the Ethical and Animal Welfare Committee of the *Universitat Autònoma de Barcelona*, in accordance with the guidelines of the Good Experimental Practices.

### RNA extraction and microarray hybridization

Total RNA was extracted from 100 mg tissue using the RiboPure™ kit (Ambion, Austin, USA) according to the manufacturer's protocol. RNA was quantified with the NanoDrop ND-1000 spectrophotometer (NanoDrop Technologies, Wilmington, USA) and the RNA integrity was assessed by Agilent Bioanalyser 2100 and RNA Nano 6000 Labchip kit (Agilent Technologies, Palo Alto, USA). Due to high variation in concentrations of the total RNA obtained in different tissues, all samples were concentrated and cleaned using the RNAeasy MiniElute Cleanup kit (Qiagen, Basel, Switzerland) obtaining final concentrations between 500 and 1000 ng/μl.

A total of 64 microarrays (4 animals × 16 tissues) were hybridized and scanned at the *Institut de Recerca Hospital Universitari Vall d'Hebron *(Barcelona, Spain). Briefly, the cDNA synthesis was undertaken with 5 μg of total RNA, labelled with biotin and hybridized to individual high-density oligonucleotide microarray chips (GeneChip^® ^Porcine) from Affymetrix (Santa Clara CA) containing a total of 23,937 probe sets (23,256 transcripts), representing 20,201 *Sus scrofa *genes, 11,265 of these genes were annotated by Tsai et al. (2006). The hybridization was done according to Affymetrix standard protocols and microarray expression data were generated with GeneChip Operating Software (GCOS). As the annotation provided by the manufacturer is not too detailed, the results in this work are based in the annotation developed by [26]. The complete data set, both RMA and original CEL files, are available at Gene Expression Omnibus (GEO) under accession number GSE10898.

### Quantitative RT-PCR

We used quantitative real time PCR (QRT-PCR) to validate differential expression between sexes of one of the probes (Ssc.4897.1.A1_at) annotated as CLOCK gene [26]. Expression was analyzed in backfat tissue from 27 pigs, 14 females and 13 males, pertaining to five breeds: Large White, Landrace, Duroc, Iberian and a composite line. We used the ABBI PRISM 7900 Sequence Detection System in combination of SYBR Green chemistry (Applied Biosystems, Inc., Foster City, CA). *S. scrofa *Beta-2-microglobulin (GenBank accession number NM_213978.1) was used as endogenous control. Primers were designed using the PrimerExpress 2.0 software (Applied Biosystems, Inc., Foster City, CA) and are shown in additional file 3. The PCR amplicons were 96 bp and 108 bp long for the Ssc.4897.1.A1_at and microglobulin genes, respectively.

We used the 2-^ΔΔCT ^method for relative quantification (RQ) of gene expression [36], a comparative technique in which a target gene is normalized to an endogenous control and relative to a calibrator sample. This method requires the target and endogenous PCR efficiencies to be nearly to equal. Thus, we performed a validation experiment and we plotted the log input amount of cDNA (dilutions of 1:20, 1:200, 1:2000, 1:20000 of a back fat tissue cDNA sample) versus de ΔCt, obtaining an absolute slope of 0.0273 (<0.1) thus permitting the use of the 2-^ΔΔCT ^method for relative quantification. The High Capacity cDNA Transcription Kit (Applied Biosystems, Inc., Foster City, CA) was used to synthesize cDNA from 1 μg of backfat tissue RNA following the manufacturer's instructions. PCR amplifications were performed in a total volume of 20 μl containing 5 μl of cDNA sample diluted 1:2000 or 1:20. Primers were used at 300 nM each and at 600 nM each for Ssc.4897.1.A1_at and microglobulin genes, respectively. Each sample was analyzed by triplicate. The thermal cycle was: 10 min at 95°C and 40 cycles of 15 sec at 95°C and 1 min at 60°C. A dissociation curve was performed in order to assess that there were not primer dimer formation. The sample of lowest expression level was used as calibrator

### Data processing and statistical analysis

Quality control of CEL files was done with the *Affy *package of bioconductor [37]: RNA degradation and the raw data distribution were ascertained. All CEL files were normalized simultaneously with the RMA procedure using libaffy [38]. This software is much more efficient in terms of memory than the bioconductor application. After RMA processing, data were log-transformed for further analysis.

An initial visual appraisal of the data was carried out drawing neighbor – joining (NJ) and UPGMA trees with Mega 4.0 [39]. The pairwise distance used was 1 - *r*^2^, where *r *is the correlation coefficient across all pairs of probe levels between pairs of samples. To gain further insight, we relied on mixed model methods. These have been long being used in Animal Breeding [31], and have been advocated more recently to analyze microarray data, mainly in the context of cDNA (two color) microarray [40]. We fitted several mixed models to the data. The most basic model,

*y*_*ijkg *_= *Tissue*_*i *_+ *Breed*_*j *_+ *Sex*_*k *_+ *Probe*_*g *_+ *e*_*ijkg*_,

was used for initial exploratory analysis. Above *y*_*gijk *_refers to the expression level of the g-th *Probe *(*g *= 1, 23937) at i-th tissue (*i *= 1, 16) from animal of breed *j *(*j *= 1, 2, *i.e*., Large White and Iberian) and sex k (*k *= 1, 2 or male and female). Note that a given gene may be represented by more than one probe. However, different probes of the same gene can behave differently due to at least two reasons: alternative splicing and poor annotation. Thus, here we used the probe rather than the annotated gene in the model. Preliminary studies (results not shown) shown that including the probe rather than the gene explained a larger part of the variance. Nevertheless, we refer to differentially expressed gene to mean the gene (if known) corresponding to the probe that shows a significant differential hybridization. All *Tissue*, *Breed *and *Sex *were treated as fixed effects, whereas *Probe *was random with variance $\sigma_{P}^{2}$. Next, we evaluated the relevance of interaction probe × tissue, probe × breed and probe × sex by comparing the following models against model (1):

*y*_*ijkg *_= *Tissue*_*i *_+ *Breed*_*j *_+ *Sex*_*k *_+ *Probe*_*g *_+ *Probe*_*g *_× *Tissue*_*i *_*+e*_*ijkg*_,

*y*_*ijkg *_= *Tissue*_*i *_+ *Breed*_*j *_+ *Sex*_*k *_+ *Probe*_*g *_+ *Probe*_*g *_× *Breed*_*j *_*+e*_*ijkg*_,

and

*y*_*ijkg *_= *Tissue*_*i *_+ *Breed*_*j *_+ *Sex*_*k *_+ *Probe*_*g *_+ *Probe*_*g *_× *Sex*_*k *_*+ e*_*ijkg *_.

All interactions above were treated as random. The ratio of variances $h_{PT}^{2}=\sigma_{PT}^{2}/\text{(}\sigma_{PT}^{2}+\sigma_{P}^{2}+\sigma_{\text{e}}^{2})$, $h_{PB}^{2}=\sigma_{PB}^{2}/\text{(}\sigma_{PB}^{2}+\sigma_{P}^{2}+\sigma_{\text{e}}^{2})$ and $h_{PS}^{2}=\sigma_{PS}^{2}/\text{(}\sigma_{PS}^{2}+\sigma_{P}^{2}+\sigma_{\text{e}}^{2})$ measure the global relevance of differential gene expression across tissues, breeds and sexes, respectively. Analyses were carried out with Qxpak [41] and VCE [42]on a Linux PC.

We also explored the biometric relationship between tissues. The average distance between tissues was obtained from 1-$r_{T}^{2}$, where $r_{T}^{2}$ between tissues *i *and *j *is the squared correlation across probes between the *Probe*_*g *_× *Tissue*_*i *_and *Probe*_*g *_× *Tissue*_*j *_solutions obtained from model (2). Again, NJ-trees were drawn with Mega 4 to visualize the results. An additional measure of distance between tissues can be provided by the number of extreme probes that separated a given tissue (or a group of tissues) from the rest. An extreme probe for the i-th tissue was defined as a probe for which all four mRNA levels of the i-th tissue were either smaller or larger than the remaining 60 observations pertaining to the remaining 15 tissues. The same procedure applies when Simulations showed that the probability of having such an arrangement was very small (P ~10^-4^) for random normal variates.

Further, we examined the effects of breed and sex on gene expression. To do that, we computed the z-score, defined as the standardized difference of gene expression predictions between breeds ($z_{g}^{b}$) or between sexes ($z_{g}^{s}$), *i.e*., for gene *g *$z_{g}^{b}=\frac{PB_{g1}-PB_{g2}\;}{\sigma_{\Delta B}}$ with subscript 1 referring to male and 2 to female, and $z_{g}^{s}=\frac{PS_{g1}-PS_{g2}}{\sigma_{\Delta S}}$ with subscript 1 referring to Large White and 2 to Iberian, where *PB*_*gj *_is the prediction of the interaction between probe *g *and breed *j *(*Probe*_*g *_× *Breed*_*j*_) obtained from model (3), and *σ*_Δ _is the standard deviation of the numerator. Similar notation applies to $z_{g}^{s}$. Once the probes were ranked by their breed or sex absolute scores, we selected those with a false discovery rate (FDR) of less than 5% following the standard procedure [43]. P-values of the z-scores were obtained assuming a normal distribution with SD = 1. We performed a hierarchical cluster analyses of the microarrays using the significant (FDR < 0.05) probes for either breed or sex with Cluster 3.0 [17]. The metrics employed was uncentered correlation with the complete linkage option. Results were visualized with Java TreeView 1.1 [44].

Note that all data available were utilized simultaneously in the mixed model analyses just presented,*i.e*., 23,937 probes × 16 tissues × 4 animals ~1.5 million records. This implies that any solution in models (1 – 4) was estimated taking into account all information available. Thus, all estimates should have high accuracy, provided that the models adjust to the data. We discuss this issue in the results and discussion section below.

### Functional analyses

We obtained the gene ontology (GO) process using the onto-express platform [25]. This platform allows to compare automatically the frequency in GO classes in all genes with known annotation in the whole microarray *vs*. GO class frequencies in a target lits, e.g., the most differentially expressed genes between sexes or breeds or extreme genes in embryonic layers. False discovery rates are reported.

## Abbreviations

FDR: False Discovery Rate; GO: Gene Ontology; IB: Iberian pig breed; LW: Large White pig breed; NJ: Neighbor – Joining; RMA: Robust Multiarray Average; QRT-PCR: Real Time Quantitative Reverse Transcription Polymerase Chain reaction; UPGMA: Unweighted Pair-Group Method with Arithmetic Mean.

## Authors' contributions

MPE conceived the research. MPE, MLB and JMF supervised research, all carried out the research. ALJF and MPE analyzed the data. ALJF and MPE wrote the first version of the manuscript.

## Data availability

The data used in this study have been deposited in GEO under accession numbers GSE10898.

## Supplementary Material

Additional file 1 List of extreme probes for each of the tissues grouped according to the three embryonic layers. Ectoderm tissues were olfactory bulb, both hypophyses, pineal gland and hypothalamus; mesoderm comprised fat and muscle tissues; and endoderm, liver, stomach and ileum. An extreme probe for a given group was defined as one where all expression values in the tissue group were either higher or lower than the values in the rest of tissues (Material and Methods). The marginal difference is the difference between the maximum value of the extreme probe in the group of tissues under consideration and the minimum value in the rest of tissues (when marginal difference is negative) or between the minimum value in the group and the maximum value in the rest of tissues (a positive difference). Thus, a negative marginal diference means that the gene is underexpressed; a positive marginal difference, overexpressed. There are two sets of columns for negative and positive marginal differences, respectively. Click here for file

Additional file 2 List of extreme genes for each of the 16 tissues studied. Notation is identical to that in additional file 1. Click here for file

Additional file 3 Primers used for QRT-PCR. Click here for file
