# Supplementary material for: Transcriptome architecture across tissues in the pig
Source: BMC Genomics. 2008 Apr 16;9:173. doi: 10.1186/1471-2164-9-173 (PMC2335121; doi:10.1186/1471-2164-9-173)
Supplement: Additional file 3 — Primers used for QRT-PCR. [file 1471-2164-9-173-S3.doc]

| Primer name | Sequence |
| --- | --- |
| sc.4897.1.A1_at Fw | 5’-TTCCTTATGCCAGACCATGCT-3’ |
| sc.4897.1.A1_at Rv | 5’-ATCCAACTCCAGTCCAGATCTTG-3' |
| β-2-microglobulin Fw | 5’-ACCTTCTGGTCCACACTGAGTTC-3’ |
| β-2-microglobulin Rv | 5’-GGTCTCGATCCCACTTAACTATCTTG-3’ |
